# Albert Camus – A Psychobiographical Approach in Times of Covid-19

**DOI:** 10.3389/fpsyg.2021.644579

**Published:** 2021-03-16

**Authors:** Claude-Hélène Mayer

**Affiliations:** ^1^Department of Industrial Psychology and People Management, University of Johannesburg, Johannesburg, South Africa; ^2^Europa University Viadrina, Frankfurt, Germany

**Keywords:** Albert Camus, psychobiography, existentialist theories, terror management theory, PP20, transcendence, best practices, COVID-19

## Abstract

Albert Camus (1913–1960) stands as one of the famous pioneers in the French history of existentialism. He was a novelist, political activist, essayist and editor, as well as a journalist and playwright. Although he was described as philosopher, he often denied this ascription. Through his professional and creative expressions, Camus focused on questions of existentialism, the aspect of the human fate, and meaning in life, death and suicide. These existential questions have experienced a strong revival during the Covid-19 occurrence. This psychobiographical approach aims at understanding Albert Camus' life and work in the context of the terror management theory of Becker and Wong's 4 pillars of PP2.0 theory, namely virtue, meaning, resilience and well-being. Both theories have gained importance during the pandemic. Based on the findings of the research study, implications for future research in the context of the pandemic are given. Finally, this article provides recommendations and best practices on how to approach the Covid-19 pandemic from a terror management theory and PP2.0 perspective in the light of Albert Camus' philosophy. The contribution of this psychobiography is two-fold: first, it expands psychobiographical research on Albert Camus from absurdist and existentialist theories and thereby expands the theoretical framework of psychobiographies. Second, it aims at strengthening the importance of theoretical psychobiographical investigations and their application in real-world scenarios to address complex contemporary challenges on the basis of existentialist positive psychology theories.

*Life is nothing until it is lived*.

*It is we who give it meaning*,

and value is nothing more

*than the meaning we give it*.

Jean Paul Sartre

## Introduction

In the dawn of Covid-19 and the realization of the need for new approaches to deal with challenging life situations, role models are needed to respond to the question of how to deal with uncertainty, loneliness, defining a meaningful life, and collective and transformational ways of coping (Rodríguez-Rey et al., 2019; De Jong et al., 2020; Wong, 2020a,b; World Economic Forum, 2020). The suffering and pain experienced in daily life by individuals (Frankl, 1959) needs to be understood within the context of greater, global meaning-making (Baumeister, 1991) and the transformation of stressful events (Park, 2010). Recent research has shown that health anxiety, health-related life events and somatization during Covid-19 can increase chronic pain (Chaturvedi, 2020) as well as psychological suffering (Pietrabissa and Simpson, 2020; Walsh, 2020). Numerous scientists have emphasized that coping strategies, resilience and transformation of meaning-making are needed to deal with Covid-19 constructively (Bland, 2020; Rettie and Daniels, 2020). Bland (2020, p. 710) has recently pointed out that Covid-19 provides a chance for individuals and humanity to “[press] the reset button by prompting pause and reflection on habitual patterns and serving as an ‘urgent experience’ with the potential to spark revitalizing intentionality.” Drawing on existential–humanistic theorizing, it is suggested that dialectical worldview and paradoxes can be transcended by confronting positive and negative aspects which are simplistically overemphasized (Bland, 2020). The author (Bland, 2020) highlights that Covid-19 can provide opportunity for creativity and “collective co-creating of a cultural narrative involving evolution toward enhanced senses of consciousness and caring.” This is particularly true for any crisis which humankind has to go through. According to Yalom (1980, p. 31), the experience of a crisis is an “urgent experience” which can support reflection, revitalization and any kind of intentional change. Existentialist perspectives have emphasized four dialectical aspects in life which need attention and which humans need to respond to: life/death, community/isolation, freedom/determinism, and meaning/absurdity (Greening, 1992). According to Greening (1992), humans create mental health when they are able to respond to all of these four aspects through acceptance and creativity.

In this article, the author explores two of Greening's (1992) existential givens, namely *life/death* and *meaning/absurdity* through a psychobiographical approach which takes the terror management theory (TMT) of Becker and Wong's 4 pillars of PP2.0 theory, into consideration. The selected existential givens (Greenberg et al., 1992) are explored in depth through the application of these two theories in analyzing Albert Camus' life and work. Both of the theories have gained importance through the pandemic (Wong, 2020a,b; Krasovska et al., 2021). Based on the findings of the research study, implications for future research in the context of the pandemic are given.

Albert Camus (1913–1960) is one of the famous pioneers in the French history of existentialism. He was a novelist, political activist, essayist and editor, as well as a journalist and playwright (Aronson, 2017). Although he was described as philosopher and existentialist, he often denied these ascriptions (Arendt, 1946; Sherman, 2009; Sharpe, 2015). During his short life, Camus dealt with questions of life and death, absurdity and meaning and presented these topics in his novels, essays and writings (Arendt, 1946; Aronson, 2017). Through his professional and creative expressions, Camus focused further on questions of existentialism, the aspect of the human fate, meaning in life in the face of death, as well as suicide (Aronson, 2017). These existential questions have experienced a strong revival during the start of the new century (Hayden, 2013) and again during Covid-19 (Walsh, 2020; Wong, 2020a,b). According to Walsh (2020), people in the Covid-19 pandemic have to cope with multiple losses, such as complicated and traumatic deaths, isolation and loss of physical contact, loss of jobs, livelihoods, and financial security, and the loss of hopes and dreams, accompanied by shattered assumptions. They have to overcome human vulnerability and toxic interdependence by being creative and building resilience and hope. Bland (2020, p. 712) suggests that this positive, healthy state can best be reached when individuals are “constructively confronting, creatively responding to, and transcending the challenge by embracing its non-duality,” thereby neither overemphasizing its positive nor its negative aspects.

This study's primary aim is to explore the life and work of **Albert Camus** with special regard to questions of how to view life and death and how to lead a meaningful life in times of crises, absurdity, suffering and pain through this psychobiographical investigation. By doing so, this study adds, on the one hand, to fostering psychobiographical work on French (-Algerian) philosophers, authors and journalists and, on the other hand, explores topics of existential positive psychology (PP2.0) across the lifespan of an extraordinary individual. On a third note, this psychobiography supports the previously emphasized hypothesis (Mayer and Fouché, 2021) that psychobiographies should be used not only to explore the life and creative works of extraordinary individuals and expand theories and research methodologies, but also to use the quintessence of an extraordinary life's knowledge to strengthen constructive approaches to deal with life's dilemmas (Mayer and Fouché, 2021) such as suffering, pain, flourishing and self-development, and to use absurdity and death as inspirational forces and turning points (Yalom, 1980) to lead a meaningful life (Van Tongeren and Showalter Van Tongeren, 2020).

This psychobiographical investigation is also used to contribute to finding sustainable solutions to deal with extreme (global) challenges, humankind's crises and disasters in the light of extraordinary approaches in psychobiographical perspectives. It is argued here that this potential of psychobiographical research has not yet been exploited to find best practices and practical applications to sustainably resolve humankind's challenges in their search for meaning and existential core concerns. Therefore, individual solutions found in psychobiographies can act as role models for the readers of these psychobiographies. Finally, this article provides recommendations and best practices on how to approach the Covid-19 pandemic from an existentialist perspective, using the TMT and PP2.0 theories in the light of Albert Camus' philosophical approaches, to provide new and original perspectives in these challenging times of the pandemic.

## Ernest Becker's Terror Management Theory (TMT)

Becker (1973) developed the terror management theory (TMT) by exploring the question of how human beings can develop meaning in existential conditions to cope with life's challenges (Barrett, 1958; Pyszczynski et al., 2015). A special focus of TMT lies on the impact of death and life's meaning (Yalom, 1980; Steger, 2012). It has been pointed out that much of human behavior is driven by the fear of death—which is extensively triggered by the recent Covid-19 pandemic (Menzies and Menzies, 2020).

TMT is based on the assumption that human beings foundationally fear death; consequently, death avoidance is anchored in concepts such as sustainability, self-esteem and self-worth (Routledge et al., 2010; Van Kessel et al., 2020). The anxiety to die is overcome by believing in the meaningfulness of one's life, positive and peaceful relationships and uncertainty avoidance (Becker, 1973; Pyszczynski et al., 2015). Through the denial of death, humans deal with their death anxiety and the higher the self-esteem and the better the close relationships, the less the death anxiety (Juhl, 2019). The same is true for values and socio-cultural belief and assumptions: the stronger one's own values, the lower the death awareness (Schimel et al., 2007; Greenberg and Arndt, 2012). Greenberg and Arndt (2012, p. 405) observe that TMT originally aimed to show that “people have difficulty getting along with those who are different and that people have [a] trenchant need to feel good about themselves.” Both of these aspects buffer against fear of death and defend against thoughts and fears of mortality (Greenberg and Arndt, 2012).

Further, in TMT, there is an ongoing debate about the question whether death anxiety is positive or negative (Vail et al., 2012; Van Kessel et al., 2020). It might be experienced as positive, enriching and impactful when the experience and awareness of death leads to an increase in meaning in life through the strengthening of intrinsically motivated goals (Vail et al., 2012). Fear of death might, however, also potentially lead to disruption of the experience of meaning in life, while the experience of meaning again might lead to a constructive way of coping and dealing with death in a mindful and aware way (Heine et al., 2006). Yalom (1980, p. 33) has previously noted that life is lived best when humans become aware of and embrace the reality of death, since the realization of one's own mortality may provide a “turning point” in one's life which will be followed by an extraordinary change.

## Paul Wong's Positive Psychology (PP2.0) In the Context of COVID-19

Over the past decade Wong (2020a,b) has developed the existential positive psychology (PP2.0) approach which provides coping strategies and resources to deal with loss, fear of death, disappointment, anxiety, and despair.

While the first positive psychology (PP1.0) movement pioneered by Seligman (Peterson and Seligman, 2004) mainly focused on the positive aspects in life, Wong (2011; 2019; 2020a; 2020b) emphasizes that to increase well-being and mental health and life's worth, individuals need to accept that life involves pain and suffering and transform these challenges of life to create sustainable mental health and well-being (Wong, 2011). Wong's (2020a,b) perspective supports those of Greening (1992) and Bland (2020) in that existential givens such as life/death and meaning/absurdity need to be responded to through acceptance and creativity. According to Wong (2011), this acceptance is only reached when pain, suffering and negativities are explored, experienced and integrated. Only with their integration can they be altered toward becoming a health resource with a positive impact (Mayer and Vanderheiden, 2019). According to Hogan (2020) they can become key drivers of positive transformation in collaborative groups, providing positive collective responses to commonly shared problems. Collective empowerment, solidarity and collective intelligence are additional drivers to transform the negative into the positive (Hogan, 2020). This means that the negative and the positive aspects are being integrated and thereby transformed in all subdomains in positive psychology (Wong, 2020a,b). PP2.0 does not see the acceptance of suffering and the enhancement of happiness and well-being as independent endeavors, as was originally thought in the classical positive psychology movement, but as integrated (Wong and Worth, 2017). According to Wong (2011), PP2.0 is built on the 4 major pillars of virtue, meaning, resilience and well-being. For the study at hand, the pillar of meaning is of major importance, being reflected in the four givens—life/death, and meaning/absurdity (Greening, 1992). Meaning is viewed as an important part of well-being, happiness and part of a good life (Wong, 2015, 2020a,b). Meaning (Wong, 2012) is explained in terms of the “PURE” model, referring to the components of Purpose (overall direction, life goal, core values, framework for decisions), Understanding (self-identity, worldview, the pursuit of self-understanding and self-knowledge), Responsible action (ethical decisions and actions, well-being of others) and Enjoyment and evaluation (positive feelings, evaluation and reevaluation, corrections, motivation for positive change).

## Research Methodology

This study uses a case study design to explore the life and work of Albert Camus through the lens of the presented theories. The research paradigm is qualitative in nature and uses a hermeneutical-interpretative research approach (Creswell, 2013).

### The Sample

The sample is purposefully selected (Shaheen and Pradhan, 2019) and focuses on one famous and extraordinary French-Algerian philosopher who explored existentialism and who published plays, novels and essays on existential questions, life and death, meaning and absurdity of life (Camus, 1938, 1942a,b). In particular, Camus wrote a novel on life in the times of a crisis, *La Peste*, narrating an allegorical account of the fight against an epidemic in Oran, a town in Algeria. (Camus, 1947). Another famous work is Camus (1956); the English edition is called “The Fall” and, as a philosophical novel, the book deals with the self-reflections of a man. These self-reflections on life are presented as monologs which are held by the protagonist, a wealthy Parisian lawyer, in front of a stranger. The monologs are a kind of confession and deal with issues of innocence, imprisonment, non-existence and truth and finally, the fall from grace. The novel, in its deeper sense, deals with the question of values, humankind's evil and explanations of how humans believe in their own kindness which covers their selfishness, egocentric and narcissism in a most unconscious manner. Only when humans realize the underlying shadowy mindsets, do they experience crisis, which, in the case of the novel's protagonist, leads to his self-enhanced fall and ruining of his reputation.

In fact, all of Camus' works deal with existential values and questions within and beyond humanism, circling around the struggle with individual and societal crisis (Camus, 1942a,b, 1947), but also the public and the private face and masks people wear.

### The Author's Approach to Data Collection, Analysis, and Interpretation

The author developed interest in Camus and his life, as well as other French philosophers and existentialists, during the 1990's. She used to sit in a French Crêperie, reflecting upon existentialism, human values and meaning in life. Since then, the interest in Camus never subsided and became vividly alive during the past year of the pandemic in which the author experienced thoughts and reflections on human crisis which she had experienced reading Camus in the 1990s. She then started to review Camus once more, his work and life during the first year of the pandemic and reflected on her “new” understanding of Camus in comparison to her understanding during the 1990s.

During the process of research, the author spent about 1 year reading and re-reading Camus' pieces, the primary and secondary data were collected. Several authors have written on Camus' biography and his literature [see Bloom (1989), Brée (1961), Brée (1962), Cruickshank (1959), Cruickshank (2020), Foley (2008), Hughes (2007), Lottman (1979), Margerrison et al. (2008), McBride (1992), O'Brien (1970), Sartre (1965), Srigley (2011), Todd (1997), Zaretsky (2013)], including primary data, for example autobiographies, and secondary data, for example biographies, interviews, and articles. The process of exploring Camus and his work started in the beginning of 1990s and experienced a peak in 2020 when the pandemic hit the world. Therefore, the relationship of the researcher to the researched is a long-standing relationship and based on a deep, longitudinal interest and reflection.

A five-step process of content analysis (Terre Blanche et al., 2006) was used to analyse the data. The steps of analysis are familiarization and immersion, inducing themes, coding, elaboration, and interpretation and checking. The period of familarisation and immersion in the data started in the 1990s and experienced a peak and expansion in 2020, when primary data were re-read and secondary data were consulted to explore Camus' approach to life and death, meaning and absurdity in more depth within the context of Covid-19. His writings were used to improve the understanding of the contemporary crisis and for self-transcending the situation of the global pandemic in a more conscious and informed way. In the following different themes emerged and theories were explored which corresponded with the themes. The themes included life and death, meaning and absurdity and included codes, such as well-being, happiness, suicide, meaninglessness, irrationality, life worth living, joy, resilience etc. Primary and secondary data were coded and theories were applied while data were read by using different theoretical lenses. Finally, a decision was made to explore Camus through the lens of Greening's (1992) existential givens of life/death and meaning/absurdity, Becker's TMT and Wong's PP2.0 theory. Findings were then interpreted and checked.

### Ethical Considerations and Limitations

In terms of ethical considerations, this study follows the usual requirements for psychobiographical studies in which data are accessed from the public domain (journals, newspapers, video clips and the like.). Data are treated with respect, and information about the subject of research is treated with fairness and consideration (Ponterotto et al., 2017). As according to Ponterotto and Reynolds (2019) this study is supposed to contribute to the learning about the lives and works of others, about human diversity and the potential of human beings. Thereby, the subject of research is explored in an ethical, respectiful, empathetic and accountable manner (Schultz, 2005; Ponterrotto, 2015) to, on the one hand, contribute to the public knowledge of Camus and on the other hand to exclude potential harm for the researched subject (Wegner, 2020).

Quality criteria were applied, such as trustworthiness and rigor. Triangulation of data, theories and methodology was also applied.

As is true for every study, this study comes with limitations. It is limited by being a single case study which focuses on life history data of one selected extraordinary individual. The study is descriptive and ideographic and provides in-depth insights regarding one individual only. The perspective taken on Camus' life and work is focused on the TMT and the PP2.0 perspective highlighted above, aiming at exploring two existentialist givens, referring to Greening (1992). Further, only data were collected which had been published and were available in the public domain in either English, German and/or French. The author reads all 3 languages fluently and all the reading, integrating and discussion of findings were done solely by herself. No intersubjective validation processes during data collection, analysis and interpretation were carried out. Due to that self-centered, self-reflective and individual approach to the data, the findings might carry a strong subjective bias of the author which might be seen as a limitation to the study and the findings. It needs to be highlighted that the researcher is a female Generation X researcher, who was born in Germany, holding a strong interest in and passion for French philosophy, literature and culture of the twentieth century. The author did not use any assistant throughout the entire research and writing before submission to the review process of the journal. This might be seen as a limitation and a risk of a strong subjective bias with regard to the selection of units of analysis and interpretation of the data and findings.

## Findings and Discussion

In the following, the findings with regard to the life and work of Albert Camus will be presented in a thematic order and through the lens of the two selected givens of life/death and meaning/absurdity and the TMT and Wong's PP2.0 theory in the context of Covid-19.

### Life and Death

Albert Camus was born in Mondovi in French-Algeria on November 7, 1913. He grew up with his mother and grandmother in poor working-class family conditions. His mother, Catherine Hélène Camus (née Sintès), was of French-Balearic origin while his father, Lucien Camus, an agricultural worker, died in WWI in 1914 in the Battle of the Marne.

Camus spent his first years in Algiers, in the neighborhood of Belcourt. He was of the second-generation French immigrants to Algeria which was a French territory from 1830 to 1962. His paternal grandfather had moved to Algeria in search of a better life. Camus therefore belonged to the “*pied noir*,” a slang term for French immigrants who were born in French Algeria (Cruickshank, 2020). His poor living conditions in Algeria during his childhood, teenage years and young adulthood and his experience as an immigrant in Algeria influenced his life strongly.

At the age of 11 (in 1924), Camus received a sponsorship to attend a prestigious lyceum near Algiers from one of his outstanding teachers, Louis Germain, who supported his educational endeavors. In 1930, Camus contracted tuberculosis and lived with his uncle for a short period, before living on his own (Cruickshank, 2020). From then onward, Camus had to deal with a life of suffering, pain and endless difficulty often spending time in southern France where the climate contributed to his healing and well-being (Luckner, 2020).

Camus' uncle and his philosophy teacher, Jean Grenier, mentored him in studying philosophy when he became interested in Friedrich Nietzsche and the ancient Greek philosophers, such as Plotinus and St. Augustine (Cruickshank, 2020). Three years later in 1936, Camus enrolled at the University of Algiers and gained his Bachelor degree in philosophy while still recovering from tuberculosis (Cruickshank, 2020). He wrote his thesis on Plotinus and was strongly influenced by Nietzsche, Schopenhauer and Christian philosophers (Luckner, 2020).

During his thirties (1943–1953), Camus wrote several of his most popular novels and essays, such as *L'étranger* (Camus, 1942a), *Le Mythe de Sisyphe* (Camus, 1942b) and *La Peste* (Camus, 1947), referring to the topics of the absurd, human connection, meaning and death. The book *Le Mythe de Sisyphe* (The Myth of Sisyphus) was, according to Camus, directed against the French existentialists (Sherman, 2009). One of his favorite ideas was that life is absurd; accordingly, he considered the absurd as playing a core role in French existentialism (Sherman, 2009). However, he did not classify himself as an existentialist since he rejected the ideas of other existentialists, such as the contention of Sartre that there is secularity in life while seeing it as being absurd (Sherman, 2009). Camus sees the world as unreasonable and the absurdity lies within the confrontation of irrationality and humankind's wild longing and striving for clarity (Popova, 2014). Humankind and the world are only connected through absurdity. Zaretsky (2013), referencing Camus, suggests exploring the silence of the world—the non-responding universe—which contains the absurd. Only then can humankind realize that it has to respond to the question of where to find meaning and how to construct a life worth living themselves.

Camus prioritizes the idea that humans cannot and should not ascribe more meaning to death than to life (Hendricks, 2018). This attitude is also reflected in the rejection of suicide as a solution to the experience of a meaningless life and to the idea of afterlife which, according to Camus, distracts from the acceptance of meaninglessness of life and life's enjoyment during this lifetime (Hendricks, 2018). Death, however, is ultimately what keeps humans connected, since life ends for all humans with death (Popova, 2014). The TMT explores the impact of death, and in particular death anxiety, on life's meaning (Yalom, 1980; Steger, 2012). This fear of death leads humans to believe in creating a meaningful life which distracts them from their death anxiety (Becker, 1973; Pyszczynski et al., 2015). Camus, however, rejects this idea and instead believes in accepting a meaningless and absurd life and enjoying life for the simple reason of living, even without meaning (Camus, 1942b). At Camus' funeral, Jean Paul Sartre highlighted his “stubborn humanism” (Simpson, 2019) and the absurdity of his sudden death which at the same time shows his humanness and the inhumanity of life (Sartre, 1962).

The discourse of Camus (1942a,b) on life and death seems to be more fundamental and radical than that of the TMT, in the way that Camus is very clear about the absolute meaninglessness and absurdity of life. TMT accepts that humans create meaning to avoid death anxiety and death awareness and does not necessarily emphasize a fundamental meaninglessness, but recognizes that increasing death awareness may bring about powerful change (Yalom, 1980). This change is also addressed by Camus, who highlights that only through the awareness of death do humans find solidarity. Particularly through hardship and catastrophes, humans can transform and become resilient and strong (Hogan, 2020). In PP2.0, Wong (2020a,b) touches on Camus' (1947) idea that in the threat of a plague, humans can connect, create solidarity and develop resilience and coping mechanisms to overcome death anxiety, despair and fear through deep acceptance. However, one of the main characters in “La Peste” (Camus, 1947) continues to fight for his fellow human beings during the plague, no matter how hopeless and absurd the situation seems to be and how low the chances of overcoming the plague are (Gloag, 2020). Camus (1947) thereby shows how human solidarity in the face of death can create a positive collective response and can be a key driver of individuals to create empowerment and solidarity as described by Hogan (2020). In several of his works, Camus (1942a,b, 1947) touches on PP2.0 concepts such as virtue (morals and values), meaning (through inherent meaninglessness), resilience (through individual and collective action) and well-being (particularly in terms of happiness) and describes the concepts in a discursive way within the givens of life and death.

In 1957, Camus received the Nobel Prize for Literature (Cruickshank, 2020) as one of the youngest recipients ever. Camus' life ended tragically on January 4, 1960, at the age of 46. He had spent time with his friends and family in rural France and was on his way back to Paris with his friend and publisher, Michel Gallimard. Gallimard was driving the car when they crashed into a tree. There have been speculations that the KGB was involved in Camus' death, because of his anti-communist views (Cantelli, 2020). Cantelli (2020) suggests that Camus was silenced by the KGB because of his campaign against the Soviet crushing of the 1956 Hungarian Revolution. However, this viewpoint remains strongly debated (Flood, 2019).

### Meaning and Absurdity

Camus is most often associated with the absurd, emphasizing that life is, *per se*, absurd and meaningless. Camus' worldviews and philosophical ideas on life and existence are non-comforting and challenging (Hendricks, 2018). In Camus' mind, life and existence in itself does not contain meaning (Solomon, 1993). Consequently, Camus' discourse on meaning can be viewed as contrary to the core of PP2.0 discourse which refers to Viktor Frankl (Wong, 2020a,b; Krasovska and Mayer, 2021), emphasizing that life in itself contains meaning.

Camus views the absurd as being in the interim space of attributing meaning to life as the “unreasonable silence” of the universe (Kuiper, 2021). Thereby, meaninglessness is experienced through alienation (Battista and Almond, 1973) and the question of meaning in life becomes a core of Camus' view on human endeavors, since “without meaning, nothing matters” (Kinnier et al., 2003, p. 106) and life without meaning is “not a life worth living” (Hendricks, 2018). According to Popova (2014), the historian Zaretsky (2013) emphasizes that the exploration of meaning and its consequences are “matters of eternal immediacy” which are addressed by Camus through a rigorous consciousness which refers to the core of human existence.

In general, Camus emphasizes that the meaning in life needs to be more important than the meaning in death and he therefore rejects suicide as a solution to a meaningless life (Hendricks, 2018). Throughout his lifetime, Camus tackled the question of what makes life meaningful, foundationally highlighting that life in itself does not have meaning and that any approach to make life meaningful is impossible since humans will never be satisfied with their response to it (Hendricks, 2018).

If there is any possible created meaning in life at all, according to Camus, this meaning lies in the value of the individual human life which must influence politics rather than being influenced by ideology (Zaretsky, 2013). Zaretsky (2013) suggests that the reading of Camus' writings contributes to observation and self-reflection, which might lead the reader to becoming “a more thoughtful observer of our own lives.” This contributes to constructing meaning in life when individuals realize the injustice in the world, and struggle against the injustice, which then makes life worth living (Zaretsky, 2013). According to Kinnier et al. (2003), Camus is not only driven by the idea of meaning of life—which is related to political struggles—but also in general by the idea that life has no cosmic meaning. Here, Kinnier et al. (2003) drawerexcvb on the idea of Yalom (1980) of a cosmic meaning being divinely inspired. Instead, Camus assumes that humans can create their own meanings, independent of a divine and cosmic meaning (Camus, 1942b) since “any meaning that is based on the universe will end in a disaster” (Hendricks, 2018).

Finding the meaning in life and creating it is based on the perspective that humans need to find and create meaning themselves through courage. This courage needs to be used to “face the meaningless abyss and take responsibility for creating meaning out of the chaos” (Kinnier et al., 2003, p. 107). According to Camus, any attribution of meaning through philosophy, science, the society or religion will always lead to realizing the absurdity of life (Hendricks, 2018) and therefore courage is needed to encounter this absurdity.

Based on his approach to philosophy, his novels, politics, morality, Camus is still a particularly controversially discussed philosopher (Kirsch, 2017). For Camus, “intellectual honesty” is extremely important in the context of questioning how to live life and how to make it meaningful. He reflects on two levels of ethics, namely normative and meta-ethics (Sherman, 2009). Normative ethics deal with questions concerning the principles and rules by which people should live, drawing on ideas about right and wrong. Adhering to the right things in life gives life meaning. Meta-ethics deal with “the ethics of the overall standing of the moral enterprise itself,” such as its basic nature, its viability and its justification (Sherman, 2009). So, from Camus' perspective, life needs to be viewed as an ethical endeavor on different levels. Within the context of the question of life and its meaningfulness, Camus extensively reflects on absurdity, silence, revolt and fidelity, as connected aspects of existentialist discourses.

Haber (2019) argues that Camus' lyrical metaphors in his essays and novels, which deal with the vast unknowns of humankind, support individuals to humanize and to articulate challenging uncertainties. The metaphor of prisons in Camus' novels, for example, is viewed as corresponding to subjective imprisonment by enslaving thought-systems, from which people aim to liberate themselves to create meaning. He declared the world as being a prison which is often self-imposed by unawareness and unconsciousness (Camus, 1942a). During times of plagues (Camus, 1947), humans become prisoners of the plague and of their mindset regarding how to deal with it. However, their rebellion against the plague, and their idea of not giving in although the situation is hopeless, provide the idea of Camus' hope and optimism in times of hopelessness, his fight for moral values, humanism and the idea to fight for human connection and the human community while there is a seemingly meaningless choice between death and death (Camus, 1947; Sparknotes, 2020).

Camus stands for values such as passion, freedom, and rebellious individuation, aiming at “giving the void its colors” (Camus, 1942b; Haber, 2019). By doing so, Camus highlights the importance of facing “the absurd finitude” by fostering creativity, passion and engagement and the possibility to co-create spaces (Haber, 2019). “The Absurd” is the concept Camus is often associated with, while simultaneously opposing the idea of systematizing thought (Sherman, 2009). Camus believes that humans aim at finding meaning in life although it does not exist—and that is why they then attempt to create it (Hendricks, 2018). However, since the universe does not contribute to responding to the human quest, life is and will always be absurd and humans will always have to face absurd situations, particularly in these moments when the creation of meaning-making fails. Camus does not judge the described and inherent meaninglessness as negative, but suggests that humans need to understand the meaninglessness and absurdity. Based on this, humans can experience themselves as “being fully alive” and enjoying life (Hendricks, 2018). Camus (1942b) contends that after acknowledging the absurd and the meaningless, three important consequences need to follow, namely revolt, freedom, and passion.

Based on the notebooks of Camus (2008), Zaretsky (2013) points out that Camus was aware that absurdity could hit humans at any time, but that beauty and happiness could do the same. Often, humans would just not realize that they were happy and that they experienced beauty until the point where they “were not anymore” (Popova, 2014). At the same time, Camus expressed his idea that people hide their happiness since it is associated with laziness, but that only happy and strong people can help those who are not. In this sense, happiness and the eradication of obstacles are important steps to explore life's meaning, to live with presence and in a happy state, despite the fact that humans are impermanent.

## The Meaning of Camus In Times of COVID-19

As discussed above, several authors and scientists call for new reflections on the meaning of life and how to cope with the pandemic in a transformational and healthy way (Rodríguez-Rey et al., 2019; De Jong et al., 2020; Wong, 2020a,b). In this part of the article, the meaning of Camus' life and work is reflected with regard to the Covid-19 pandemic.

In 2020, the World Economic Forum suggests that Camus' (1942a) *La Peste* is one of the most important books to read with regard to the Covid-19 outbreak. In the context of the life and work of Camus, and in the wake of the Covid-19 pandemic, Chowdhury (2020) highlights that humankind has to realize that epidemics have occurred at different times and always present themselves in similar ways, requiring responses to evoking questions, such as: How do we respond to the “walking nightmare”? How do we deal with the reoccurring situation in the light of an indifferent universe, a godless society? These questions touch on Frankl (1959) premise that meaning in life needs a conscious understanding through suffering and pain. Chowdhury (2020) points out that during these kinds of pandemic crises, existential approaches and paradigms emerge forcefully to deal with the absurdity of life and death and its impact on daily life situations. As highlighted by Park (2010), crises call for the transformation of these stressful events which cause increased pain and psychological suffering (Pietrabissa and Simpson, 2020; Walsh, 2020). Camus (1942a) contends that the “urgent experience” of the pandemic requires reflection on habitual patterns, consciousness and caring (Yalom, 1980; Bland, 2020), as described in *La Peste* (Camus, 1942a).

Greening's (1992) existential givens of life/death and meaning/absurdity become urgent in the wake of the new realities of Covid-19 and can be reflected based on selected aspects of Camus' philosophical and existentialist approach. It needs to be highlighted here that the reflection of Camus in the context of Greening is relatively new and original and to see Camus in this light sheds new insights onto Camus' life based on a systematic theoretical matrix.

The themes of life and death, absurdity and meaning, as addressed by Camus, have increased in importance during the Covid-19 pandemic. Researchers highlight that a new focus on these topics has been created by individuals, societies and humankind finding themselves being threatened by the experience of an existential crisis during the pandemic (Ali and Lalani, 2020), being preoccupied with questions of death—as in Camus' *La Peste* (1947). Vulliamy (2015) explains that during pandemics, humankind focuses on death and becomes preoccupied with it, confronts it on an epic scale, while continuing to fight it under consideration of “ill-defined moral justice.” Ingram (2016) points out that through the constant threat of the pandemic, morality is equally threatened and confusion arises with regard to how to understand the new situation, how to deal with it and how to define meaning in the occurrence of the new threat. These aspects increase the experience of “absurdity,” according to Vulliamy (2015), and result in similarly absurd behavior.

Schnell and Krampe (2020) confirm that meaning-in-life questions have increased during the pandemic—particularly through prosocial acts and creative expressions. The creation of meaning in life can be viewed as a psychological resource, and people with high levels of meaningfulness are viewed as more hopeful, optimistic, and stress-resistant, while people with low levels of meaning are usually threatened by loss of identity, suicide, higher levels of pain and suffering and aggravated distress during the pandemic (Ali and Lalani, 2020). The authors have found that people with high levels of meaning during the pandemic are far more resilient and successfully cope with life's challenges during this time. They also handle the crisis in meaning-making much better than people with low levels of meaning (Ali and Lalani, 2020). How does Camus' worldview fit with these findings?

Camus' ideas and philosophical approaches can significantly contribute to creating meaning in life during the Covid-19 pandemic, in that although he defines life as being in itself meaningless, he advises one to enjoy “the little things” in life (Hendricks, 2018). According to Camus, life should be enjoyed despite the experience of meaninglessness, since the fact that life is meaningless is just the platform a life is based on, a mere fact that must be realized, acknowledged and dealt with while enjoying life. Camus emphasizes the importance of accepting and suffering through the pain of the realization of meaninglessness in life; he views this as a better option than to impose meaning on it (Hendricks, 2018). The meaninglessness and absurdity in life, however, should not impact on the joy in life. This suggests that it is more effective to deal with meaninglessness straightaway instead of imposing meaning on situations and then having to face the throwback into experiencing inherent meaninglessness of any life event.

Viewed through the eyes of Camus, the pandemic of Covid-19 is a random, meaningless event which evokes multiple discourses around the search for meaning, the desperate search for understanding its inherent meaning for coping with the new challenges, the disease, the pain, the suffering and the ever-present death. Based on this discourse of multiple attempts to create meaning, Camus' worldview could be to recognize and accept the fact that there is no meaning in the event of Covid-19 and that the situation needs a radical acceptance of general meaninglessness. Then life can, despite its meaninglessness, be enjoyed by focusing on “the little things,” which are in Camus' view found in the experience of nature, sport, relationships and human interaction (Hendricks, 2018). The person who can embrace lack of meaning, accept it as a given and transform the suffering and pain of this recognition into a positive mindset and attitude is, according to Camus, an “Absurd hero” (Hendricks, 2018), like Sisyphus who rolls his boulder up the mountain throughout his life with joy (Camus, 1942b). While several existentialist positive psychologists emphasize the importance of a creating of a meaningful life (Van Tongeren and Showalter Van Tongeren, 2020; Wong, 2020a,b), Camus suggests to rather accept the meaninglessness and enjoy oneself in consciousness.

In his novel *La Peste* (1947), Camus describes how “unthinkable” the plague is for the inhabitants of a small town, Oran, in Algeria. Since the residents of the time are preoccupied with themselves, their own individual lives, their TVs and their alcohol consumption, they fail to recognize the significance of the moment when the plague takes over their lives and the rats come onto the streets, from their underworld, to die in full view. Only when the next door neighbors start dying from the disease, is the unconsciousness of the residents swept away. The abstraction of death and suffering becomes a concrete experience which shows the residents that their preoccupation with themselves is absurd.

The content of the novel *La Peste* (Camus, 1947) can easily be transferred to the times of Covid-19, creating awareness and consciousness with regard to the perspectives of humankind on life and death, as well as on setting priorities with conscious care. In Covid-19 times, the plague becomes a symbol of the sleepwalkers and the unconscious innocents who follow their individual interests without realizing the impact of Covid-19 on the world. While Camus refers to elite interests, political corruption, ecological warfare and the silence of the oppositions, this view might be—under careful considerations—also applied to Covid-19 times.

The denial of the plague in Camus' novel (1947) leads to the unconscious bubbling up of evil and evokes worry, depression and irritation in the residents. However, these feelings do not help to confront the plague, but rather strengthen it, making both the sick and the healthy plague-ridden and tired. According to Curtin (2017), humans need to stand up against the plague, to oppose “the evil” with integrity and this might even work through the pain and suffering to transform it, as proposed in Wong's (2020a,b) PP2.0.

From a TMT viewpoint, Covid-19 evokes a strong reaction in humans to develop meaning in existential conditions to cope with the challenges of the time (Barrett, 1958; Pyszczynski et al., 2015) in the context of increasing death (Yalom, 1980; Steger, 2012). Through his philosophical assumptions, Camus (1947) aims at fighting back the urge to believe in meaningfulness of life to overcome death anxiety, as explained in TMT. As in *La Peste*, (Camus, 1947) people stay unconscious as long as possible to avoid a conscious, growing death anxiety (Juhl, 2019). Yalom (1980) and Camus (1942b) point out that death awareness leads to a worthful living.

Covid-19 has evoked various reactions in individuals, socio-cultural groups and on global levels and Pyszczynski et al. (2020) have emphasized that the fear of death—independent of whether one sees the virus as a major threat or a minor inconvenience—plays a huge role in reacting to the virus. The authors further highlight that the virus is a dramatic reminder of death and vulnerability. At the same time, many resources to withstand the virus have been depleted by serious lockdowns in countries all over the world and individuals have lost their jobs and income and have drifted into social isolation of different degrees (Banerjee and Rai, 2020; FitzGerald et al., 2020). The political and overall reactions to counteract Covid-19 to “flatten the curve” have minimized the availability of coping mechanisms for anxiety and threat and individuals struggle with meaning-making and self-esteem and finally with managing the “terror of death” (Pyszczynski et al., 2020). Hayes and Smith (2005) have highlighted that acceptance and commitment can help diminish the fear of death and to experience life more fully. While Wong (2020a,b) recommends that individuals and humankind need to react with acceptance and creativity to life/death and meaning/absurdity, Camus also proposes that these givens need acceptance (Camus, 1942a,b, 1947; Luckner, 2020). Only then life can be enjoyed [Luckner (2020)].

Virtue, meaning, resilience and well-being are highlighted by Wong (2020, 2011; 2012) as the four pillars of PP2.0 in times of Covid-19 to address mental health issues. Meaning is viewed as an important aspect of well-being, of happiness and as part of a good life (Wong, 2015, 2020a,b). Camus, however, does not see an inherent meaning in life, pointing instead to the need to accept the absurdity of life and noting that happiness and joy can be experienced independently of life's meaninglessness. He, however, also points out, as in the PURE-model (Wong, 2012) that Purpose, Understanding, Responsible Action and Enjoyment play a role to transform the meaninglessness and absurdity of life through radical acceptance.

## Conclusions

This study's primary aim was to explore the life and work of **Albert Camus** with special regard to questions of how to view life and death, meaning in life and absurdity in the context of TMT and PP2.0 in times of Covid-19. To respond to this aim, a psychobiographical approach was used to point out views which can contribute to deeper reflections on the four givens and to coping strategies during the pandemic.

The findings show potential responses to times of crisis with regard to Camus' worldview and the idea that existential–humanistic paradoxical approaches can be transformed and transcended by confronting positive and negative aspects of living. Enhancing consciousness and philosophical deep thoughts can support new and enhanced approaches to deal with the challenges. The findings show further that Camus' experiences—in particular his upbringing, the contextual experiences as *pied noir* and his personal contracting of tuberculosis—as urgent experiences (Yalom, 1980) supported his deep reflections, revitalisations and intentional ideas and changes in his life. These experiences also supported his creative expressions in his work and these can be viewed as a healthy response to life/death and meaning/absurdity, as presented by Greening (1992). In several of his creative works, Camus addressed the four givens extensively, often focusing preferably on the absurd which connects humans and the world through a non-responding universe. Camus generally recommends that humans should focus on and ascribe meaning to life rather than death; however, he sees that the overall meaninglessness in life needs to be recognized, acknowledged and overcome by enjoying life and thereby making the life worth living. The only meaningful ascription to death is that humans are most strongly connected through death which will end the life of all humans. To overcome death anxiety, however, Camus does not suggest searching for meaning in life, but rather proposes accepting it as meaningless and seeing the only meaning in life as that of living itself. It might be assumed that his radical view on life's absurdity could also be an unconscious strategy to overcome death anxiety, as is his fight for justice, passion, morals and life's enjoyment. Hardships, such as those experienced during Covid-19, can support humans—in the face of death—to transform and change.

Throughout his short life, Camus stood for the pillars of PP2.0 in the following way:

1. virtues—ethics, morals, and certain values such as justice, passion, empowerment and solidarity;
2. meaning—life does not include inherent meaning; it does for Wong (2020a,b) based on Frankl (1959), but Camus sees the only meaning in enjoying a meaningless life in awareness and consciousness;
3. resilience—for Camus being individually and collectively active, presenting solidarity and justice;
4. well-being—Camus particularly refers to happiness as one important aspect of well-being and to transform and overcome the meaninglessness and absurdity of life.

It can be concluded that the 4 pillars of PP2.0 are important in Camus' life and works and contribute to the concept of “a life worth living in meaninglessness.” They are strongly connected to a rigorous consciousness and the core of human existence which can increase self-reflection through observation, and meaningfulness through political struggles and selected societal values which are worth living and fighting for. However, this meaning-making needs courage, since each search for meaning evokes a confrontation with the absurd.

Particularly in Covid-19 times, when death confrontation and awareness are immediate and powerful, individuals need to free their minds and create a positive, hopeful outlook, according to Camus, which might need a “rebellious individuation” to break free by using revolt, freedom and passion.

The contribution of this study finally provides a theoretically-based applied approach of selected thoughts and insights of Camus in the context of Covid-19. It does not show Camus as an existentialist who denies meaning in life, as often proposed. It rather contributes to developing a more differentiated view on Camus: as a person who saw life as absurd and as *per se* meaningless, but also as a person who knew how to create meaning and joy in a meaningless world. With this dialectic view, the study might bring about new ideas for the reader on how to approach life and death, meaning and absurdity in times of crisis. The study further aspires to presenting Camus not only as a historical, philosophical figure, but rather as a visionary meaning-maker in times of crisis—not only by drawing on historic and metaphoric pandemics, but also in the prospect of contemporary and future pandemics. If “La Peste” was Camus' metaphor for the pandemic of the German Nazis in the 1940s in Europe, –what metaphor would Covid-19 in Camus eyes be for the world in the 2020's?

The use of the theories of TMT and PP2.0 held up what they promised for this study: they provided perspectives on Camus' life from existentialist and positive psychology stances, thereby bringing them together to what Wong (2021) calls Existential Positive Psychology. The combination of these two different approaches sheds a new light on different aspects of Camus' life and his worldviews. It expands both theoretical approaches through synergetic, interdisciplinary, and original perspectives. It contributes to support selected aspects of TMT on the one hand, while showing on the other hand that the PP2.0 perspective explores deep suffering and deep joy throughout an individual's life span. Combining both theories leaves the reader with more than one perspective and a complex, systemic view on Camus' life and meaning-making in general. Finally, this study provides individuals who suffer through the pandemic - with all its losses, deaths and meaninglessness–with the idea that there might not be any meaning in this pandemic, except that it requests a radical acceptance, as well as transcendence, new foci and new thinking toward life, joy and self-imposed meaning-making.

## Recommendations

Based on the findings of Camus' approaches to life and his works, it can be recommended on a scientific level that future research should further focus on the detailed exploration of Camus' life and work from a psychobiographical perspective and its detailed contribution to the four givens, TMT and PP2.0. This could foster a clear demarcation of the theories, and thereby contribute to deepening and extending them. Additionally, the role and perspectives of different existentialists should be explored in depth to establish how their discourses can contribute to the theories presented in this article.

In terms of (psychological) practice in times of Covid-19, Camus' insights could shed some light on perspectives of how to deal with life and death in a practical way during the pandemic. Camus' theoretical discourses might help individuals to understand, manage and cope with the pandemic's challenges, the pain and the suffering, and enable a deeper understanding and acceptance of meaning, lack of meaning, and absurdity.

## Data Availability Statement

The raw data supporting the conclusions of this article will be made available by the authors, without undue reservation.

## Author Contributions

The author confirms being the sole contributor of this work and has approved it for publication.

## Conflict of Interest

The author declares that the research was conducted in the absence of any commercial or financial relationships that could be construed as a potential conflict of interest. The reviewer JP declared a past collaboration with one of the authors C-HM to the handling editor.
